# *SPARC* aberrant methylation in idiopathic pulmonary fibrosis: an explorative study

**DOI:** 10.3389/fcell.2025.1639844

**Published:** 2025-09-03

**Authors:** Federico Pio Fabrizio, Angelo Sparaneo, Flavia Centra, Francesco Delli Muti, Paola Parente, Massimiliano Copetti, Marco Donatello Delcuratolo, Antonio Rossi, Elisa Gili, Giulio Rossi, Paolo Graziano, Lucia Anna Muscarella

**Affiliations:** ^1^ Laboratory of Oncology, Fondazione IRCCS Casa Sollievo della Sofferenza, San GiovanniRotondo, Italy; ^2^ Department of Medicine and Surgery, University of Enna “Kore”, Enna, Italy; ^3^ Unit of Pathology, Fondazione IRCCS Casa Sollievo della Sofferenza, San GiovanniRotondo, Italy; ^4^ Unit of Biostatistic, Fondazione IRCCS Casa Sollievo della Sofferenza, San GiovanniRotondo, Italy; ^5^ Unit of Oncology, Fondazione IRCCS Casa Sollievo della Sofferenza, San GiovanniRotondo, Italy; ^6^ Oncology Centre of Excellence, Therapeutic Science & Strategy Unit, IQVIA, Milan, Italy; ^7^ Department of Clinical and Experimental Medicine, University of Catania, Catania, Italy; ^8^ Department of Anatomical Pathology, Fondazione Poliambulanza, Brescia, Italy

**Keywords:** SPARC, methylation, lung diseases, IPF, molecular markers

## Abstract

**Introduction:**

Idiopathic pulmonary fibrosis (IPF) is a chronic interstitial lung disease (ILD) characterized by progressive accumulation of extracellular matrix in the lung and dysregulated activation of specific signaling pathways. Recent advances in the understanding of the biological bases of IPF identified the silencing of secreted protein acidic and rich in cysteine (SPARC) as a key modulator in the pathogenesis of IPF, although the mechanisms underlying the SPARC aberrant modulation remain to be fully elucidated.

**Methods:**

Here we investigated the aberrant methylation at the promoter gene region as a possible mechanism of SPARC deregulation in IPF. Formalin-fixed paraffin-embedded (FFPE) tissues from a cohort of 44 patients with IPF and from a control-group of 23 non-idiopathic pulmonary fibrosis (NIPF) were analyzed. DNA methylation analysis at the *SPARC* promoter region was assessed by quantitative methylation-specific PCR analysis (QMSP) and a total of 11 CpGs located in the gene promoter island were evaluated.

**Results:**

Methylation levels were found to be significantly higher (p < 0.004, Mann-Whitney test) in 44 IPF samples (methylated using the optimal cut-off 20/44, 45%) compared to NIPF surgical biopsies (methylated using the optimal cut-off 3/23, 13%). At the *in vitro* level, we observed an inverse correlation between SPARC mRNA levels and hypermethylation under 5-Aza-2′-deoxycytidine (5-Aza-CdR) treatment when a primary fibrotic cell line was treated, whereas any variations were observed treating non-fibrotic cells.

**Discussion:**

Our explorative study suggests that promoter methylation of the *SPARC* gene is linked to IPF but not to NIPF, and could represent a potential molecular marker of disease, thus warranting further investigations on larger cohorts.

## 1 Introduction

Idiopathic pulmonary fibrosis (IPF) is a prototype of chronic and fatal interstitial lung disease (ILD) associated with a radiological and histological pattern of Usual Interstitial Pneumoniae (UIP). IPF is caused by alveolar epithelium injury with an abnormal response by the adjacent mesenchymal compartments, leading to aberrant and persistent activation of tissue repair mechanism (Hewlett et al., 2018). As a result, remodeling of the lung architecture is characterized by increased deposition of extracellular matrix (ECM) proteins, leading to a progressive impairment of gas exchange with restrictive damage and respiratory failure (Herrera et al., 2018). IPF predominantly occurs in elderly patients and is associated with a poor prognosis, with a median survival of 3–5 years from the time of diagnosis (Hyldgaard et al., 2020). The pathological features of IPF include heterogeneous fibrosis, fibroblast proliferation, and parenchymal remodeling, primarily in subpleural regions (Brown et al., 2019).

A large number of accepted hypotheses attribute these characteristics to the aberrant activation of injured alveolar epithelial cells which lead to the release of inflammatory mediators. This contributes to the proliferation of resident fibroblasts, the recruitment of fibrocytes, and epithelial-mesenchymal transition (EMT) enhancement (Mutsaers et al., 2023). The biological processes underlying IPF are heterogeneous, and epidemiological studies suggested a complex interplay between genetic predisposition and environmental factors, such as aging and cigarette smoke exposure, and acquired damage responses (Luppi et al., 2021). However, and in most cases, lung fibrosis results from a maladaptive response to this intricate interplay (MacIsaac et al., 2024; Maher, 2024). Despite the advancements in the understanding of its pathophysiology, the etiology of IPF remains largely unknown, and effective treatments are limited. This challenge has driven ongoing research into novel genetic and epigenetic biomarkers (Phan et al., 2021).

Over the years, significant attention has been addressed toward understanding the role of DNA methylation in the development and progression of various respiratory disorders, including IPF. DNA methylation is a crucial biological modification and is one of the most “genetic” of the epigenetic hallmarks involved in the modulation of gene ex-pression levels in many diseases (Fabrizio and Muscarella, 2024; Ren et al., 2024). Numerous scientific studies that gene silencing by DNA hypermethylation downregulates tumor suppressors and/or antifibrotic genes in cancer and chronic lung diseases (Duan et al., 2022). As consequence, a global map of DNA methylation becomes crucial to identify specific methylation patterns and discover novel therapeutic targets, also linked to many epigenetic mechanisms, potentially involved in the IPF genesis and evolution (Valand et al., 2025).

The SPARC protein is a 32-kDa matrix-associated protein that modulates interactions between cells and the surrounding extracellular matrix (Kehlet et al., 2018). Both in non-small cell lung cancer (NSCLC) and IPF, SPARC is predominantly localized in migrating fibroblasts within fibroblastic foci and drives pathological responses by promoting ECM synthesis and turnover (Tirelli et al., 2022). Many studies have revealed a critical role for SPARC in tissue development, injury, repair and regulation of the immune response. In the lung, SPARC drives pathological responses in NSCLC and IPF by promoting microvascular remodeling and excessive deposition of ECM proteins (Wong and Sukkar, 2017). *SPARC* is a tumor suppressor gene that can be functionally inactivated through methylation (Chen et al., 2014; Fabrizio et al., 2020; Liu et al., 2018), whose link with RNA expression was widely reported in many solid tumors and also corroborated by results from large studies on gene expression profiling (Liu et al., 2018; Li et al., 2022).

Our research aimed to investigate *SPARC* promoter hypermethylation as a potential biomarker in patients affected by IPF and non-idiopathic pulmonary fibrosis (NIPF). *In vitro* experiments on fibrotic and non-fibrotic cell lines using 5-Aza-2′-deoxycytidine (5-Aza-CdR) treatment were performed to investigate the correlation between the epigenetic silencing by DNA promoter methylation and SPARC expression mRNA levels.

## 2 Materials and methods

### 2.1 Cell cultures and tissue specimens

One primary fibrotic UIP (usual interstitial pneumonia) FF24 cell line and a non-fibrotic cell line derived from a patient with pneumothorax (cell line 22) were provided by the Experimental Respiratory Medicine Laboratory (Prof. Carlo Vancheri, University of Catania). Cell lines were grown in RPMI-1640 medium (Euroclone Spa, Pero, Milan, Italy) supplemented with 10% fetal bovine serum (FBS) and 1% Penicillin/streptomycin and incubated at 37 °C with 5% CO_2_. A total of 67 specimens from patients with a clinical-histologic diagnosis of pulmonary fibrosis (44 IPF and 23 NIPF) and a subset of 11 non-fibrotic lung tissues (NFLT) were collected at the Pathology Unit of Foundation IRCCS “Casa Sollievo della Sofferenza”, San Giovanni Rotondo, Italy, in collaboration with Dr. Giulio Rossi at the Pathologic Anatomy Unit, University Hospital Policlinico, Modena, Italy.

Tissue dissection was performed under the supervision of expert pathologists, who carefully reviewed hematoxylin and eosin (H&E) stained sections from the corresponding formalin-fixed, paraffin-embedded (FFPE) blocks. For the IPF and NIPF samples, we selectively harvested DNA from regions enriched in fibrotic areas, based on histological review, rather than using whole-lung sections. Where possible, fibrotic regions were selectively microdissected to enrich for areas with active fibrosis. For NFLT, samples were obtained from morphologically normal parenchyma, distant from any pathological lesion, verified by pathologist review. This strategy aimed to reduce tissue heterogeneity and increase the specificity of our methylation profiling.

### 2.2 DNA extraction and quantification

Genomic DNA was extracted from cell lines by using the standard Phenol-Chloroform procedure, whereas DNA from FFPE specimens was obtained from GeneRead DNA FFPE Kit (Qiagen, Hilden, Germany), respectively. DNA quantification was performed using a Qubit fluorimeter (ThermoFisher Scientific, Waltham, MA, United States).

### 2.3 DNA methylation analysis

Five hundred micrograms of genomic DNA extracted from cell lines and FFPE blocks were treated with sodium bisulfite using Epitect Bisulfite kit (Qiagen, MD, United States), according to manufacturer’s instructions. Bisulfite-modified DNA is a fundamental process that serves as the template for Quantitative Methylation-Specific PCR (QMSP) to detect converted DNA (Fabrizio et al., 2017). Probe/primer sets for both *SPARC* promoter region as target gene and for the unmethylated *ACTB* promoter region as reference gene are detailed in Table 1.

**TABLE 1:** Primer/Probe sequences used for QMSP analysis for *SPARC* and *ACTB* genes.

QMSP set	Primer/Probe sequence (5′-3′)	Annealing T (°C)
*SPARC*-meth_forw	ATATTTTCGCGGTTTTTTAGA	60
*SPARC*-meth_rev	AACGACGTAAACGAAAATATCG	
*SPARC*-probe	FAM-AGCGCGTTTTGTTTGTCGTTTGTTTG-TAMRA	
*ACTB*-forw	TGGTGATGGAGGAGGTTTAGTAAGT	55
*ACTB*-rev	AACCAATAAAACCTACTCCTCCCTTAA	
*ACTB*-probe	FAM-ACCACCACCCAACACACAATAACAAACACA-TAMRA	

Meth, methylation; forw, forward; rev, reverse; QMSP, Quantitative Methylation-Specific PCR.

Serial dilutions (90–0.009 ng) of fully methylated DNA (CpGenome Universal Methylated DNA, Millipore, Chemicon) were prepared for the construction of QMSP calibration curves for both genes. 384-well plates were used to perform PCR amplification reactions in triplicate, including calibration curves for both genes, patient DNA samples, CpGenome Universal Methylated DNA as positive control, and multiple water blanks. Each reaction with a final volume of 10 μL contained 50 ng of bisulfite-modified DNA, 100 pmol/L concentrations of forward and reverse primers, 200 nM specific probe, and ROX (6-carboxy-X-rhodamine) Reference Dye, 0.6 U of platinum Taq polymerase (Invitrogen, Frederick, MD, United States), 25 mM concentrations of dNTPs (deoxynucleoside Triphosphates), and then distilled water to adjust the volume. PCR conditions were as follows: 95 °C for 2 min, followed by 50 cycles at 95 °C for 15 s and 60 °C for 1 min. ABI Prism 7,900 Sequence detection system (Applied Biosystems, Foster City, CA, United States) was used to carry out the reaction and, finally, Applied Biosystems™ Analysis Software was useful to import and analyze experiment files (software development specification, SDS 2.1.1 version, Thermo Fisher Inc., Applied Biosystems division). *SPARC* methylation levels were calculated as the average value of *SPARC* triplicates divided by the average value of *ACTB* triplicates x 1,000, as previously reported (Fabrizio et al., 2020).

### 2.4 Demethylating treatment by 5-Aza-CdR

Cell lines were cultured in a 6-well dish. 5-Aza-2′-deoxycytidine (5-Aza-CdR), an epigenetic drug that inhibits DNA methylation, was used at the working concentration of 5 μM (Sigma-Aldrich) and was added to fresh media at 24 h, 48 h and 72 h. At these time points, cells were harvested for RNA isolation to evaluate the effects of induced DNA demethylation and investigate variations in SPARC transcript levels by RT-qPCR.

### 2.5 RNA extraction and gene expression by RT-qPCR

High-quality total RNA was extracted and isolated from cell lines using TRIzol reagent (mixture of guanidine, thiocyanate and phenol) according to manufacturer’s instructions (Thermo Fisher Sc. Inc.). The SuperScript III Reverse Transcriptase kit (Thermo Fisher, Invitrogen Division, Carlsbad, CA, United States) was utilized to synthesize first-strand cDNA from RNA templates and the quantification was carried out using NanoDrop Spectrophotometer ND-1000 (Thermo Scientific). Fluorescence-based quantitative RT-PCR (RT-qPCR) was conducted using TaqMan® Gene Expression Assays (Thermo Fisher). For each reaction, 500 ng of total RNA was converted into 1 μL of cDNA, which was used as a template. TaqMan™ Gene Expression Master Mix (Thermo Fisher, Invitrogen Division, Carlsbad, CA, United States) containing AmpliTaq DNA Polymerase, Uracil-DNA glycosylase, dNTPs (with dUTP), ROX Reference, together with 250 nM of TaqMan probe, was added to the reaction. In order to construct the standard curves for real-time PCR, five plasmid dilutions (ranging from 10^6^ to 10^2^ copies) were used where cDNA was not added in the respective wells. Particularly, cDNA for SPARC and RPLPO were amplified using the TaqMan Assay and cloned into the StrataClone™ PCR Cloning Vector pSC-A (Stratagene, Milan, Italy). SPARC and RPLPO primer/probe set for gene expression were as follows and reported: Hs00234160_m1 and 4326314E (Thermo Fisher, Life Technologies), (Fabrizio et al., 2020). Amplification reactions were run on an ABI PRISM 7900HT Sequence Detection System (Thermo Fisher, Life Technologies Division). SPARC gene expression levels were normalized to the expression of the housekeeping RPLPO gene by calculating their ratio for final quantification.

### 2.6 Statistical analysis

The discriminatory power of the *SPARC* QMSP assay was evaluated by calculating the Area under the Receiver Operating Characteristics (ROC) curve (AUC). The optimal cut-off point for *SPARC* promoter methylation, which best distinguished pulmonary fibrosis tissues from NFLT, in the QMSP assay was determined at the point of the maximum Youden index (J = sensitivity + specificity – 1), which is a standard method to define optimal cut-off in diagnostic test evaluation that was applied consistently across samples to dichotomize methylation status (positive/negative), minimizing false positives and enhancing the specificity of our assay in detecting IPF-related epigenetic variations. Boxplots that illustrate *SPARC* promoter methylation levels across three different tissue types (i.e., IPF and NFLT) were also provided. Moreover, the association between *SPARC* methylation levels and patients’ histological characteristics was analyzed using Mann-Whitney U test. All statistical analyses were performed using R software (version 4.4.1). For the *in vitro* experiments, the relationship between *SPARC* promoter methylation and its expression was investigated using Student’s t-test and analyzed with GraphPad Prism 5 (GraphPad Software, Inc., La Jolla, CA, United States). All results were considered statistically significant when p is <0.05.

## 3 Results

### 3.1 Analysis performance of *SPARC* methylation assay and cut-off calculation

**FIGURE 1:**
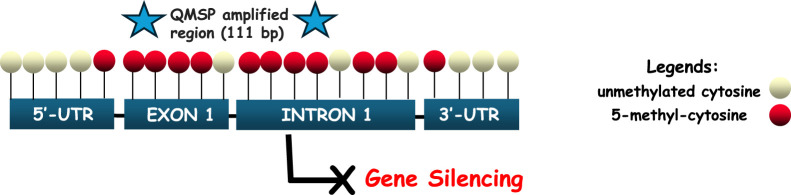
Schematic representation of the *SPARC* promoter region (5′-3′), spanning the interval sequence (−29 to +191 bp relative to the transcription starting site, TSS). QMSP amplified region covers 111 bp and is located between exon 1 and intron 1, depicted by two blue stars at either end. CpGs are marked as red circles in order to indicate the complete conversion of cytosine to uracil (5-methyl-cytosine), which can lead to the loss of gene expression. Unconverted cytosines are shown as white circles, reflecting their localization from 5′to 3′of the positive strand of the *SPARC* gene.

*SPARC* methylation status was analyzed using a specific primers/probe set targeting the CpG-rich region located at the promoter gene region, reported as mainly affected by a high variability of methylation density in cancer. The complete DNA sequence was obtained using the UCSC database (https://genome.ucsc.edu/) and the assay was designed using MethPrimer software (http://www.urogene.org/cgi-bin/methprimer2/MethPrimer.cgi) as yet reported in our previous work (Fabrizio et al., 2020). *SPARC* promoter region spans from exon 1 to intron 1 of the gene, as represented in Figure 1.

**FIGURE 2:**
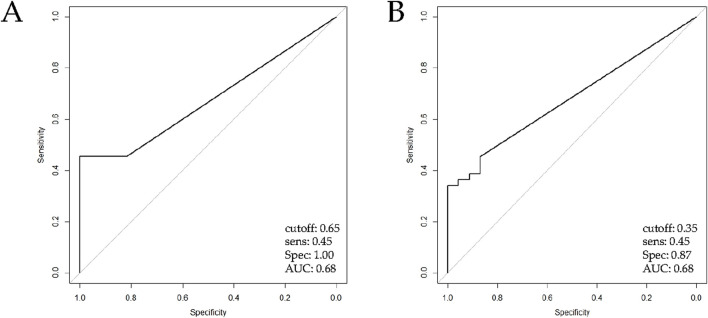
ROC curve analysis of QMSP for *SPARC* promoter methylation analysis assessed by QMSP. The ROC curve shows the sensitivity (y-axis) and specificity in discriminating **(A)** IPF patients (n = 44) versus NFLT (Mann-Whitney, p = 0.04) and **(B)** IPF patients (n = 44) versus NIPF patients (n = 23), (Mann-Whitney, p = 0.005). Abbreviations: IPF, Idiopathic pulmonary fibrosis; NFLT, non-fibrotic lung tissues; NIPF, Non-idiopathic pulmonary fibrosis.

Standard curves for *ACTB* and *SPARC* genes are shown in Supplementary Figure S1. The discriminatory power of the *SPARC* QMSP assay was assessed by estimating the AUC for NIPF and IPF subgroups using NFLT samples. When firstly compared IPF with NFLT (Mann-Whitney p-value = 0,04), an AUC of 0.68 with an optimal threshold of 0.65 was found. When applying this cut-off, methylation levels achieved a sensitivity of 45% and a specificity of 100% (Figure 2A).

Likewise, when we performed a comparison between NIPF and IPF subgroups (Mann-Whitney, p value = 0.005), ROC curve showed a threshold of 0.35 and the same AUC and sensitivity previously reported with a lower performance in specificity (87%, Figure 2B). Methylation was considered present when levels met or exceeded this threshold. ROC curve values were resumed in Supplementary Table S1.

### 3.2 *SPARC* aberrant methylation correlates with IPF condition

**FIGURE 3:**
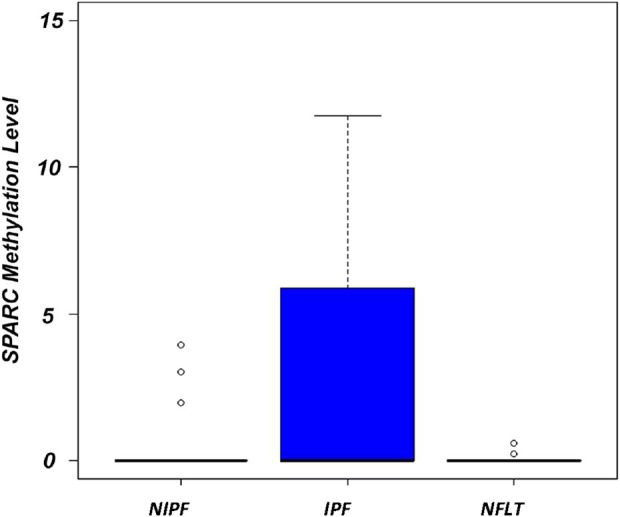
Boxplots showing the distribution of global *SPARC* promoter methylation in NIPF, IPF, NFLT. Methylation levels are expressed as (*SPARC/ACTB*)*1,000. The boxes mark the interquartile range (interval between the 25th and 75th percentile). Abbreviations: NIPF, Non-idiopathic pulmonary fibrosis; IPF, Idiopathic pulmonary fibrosis; NFLT, non-fibrotic lung tissues.

A statistically significant difference in methylation levels was observed when comparing IPF samples vs. NFLT (Mann Whitney test, p = 0.04, Supplementary Table S2) and *SPARC* hypermethylation was observed in IPF samples ranging from 0 to 119 (Mean 5,6 ± 18,1, interquartile range, IQR 0–5,8). A statistically significant difference was also observed between IPF and NIPF (Mean 0,4 ± 1,1, IQR 0–0), (Mann Whitney test, p = 0.004), (Figure 3).

Overall, DNA methylation at the *SPARC* promoter region was detected in 20 out of the 44 IPF samples (20/44, 45%). No statistically significant differences in methylation frequencies were demonstrated when NIPF samples cohort was compared with NFLT (3/23, 13%), (Mann Whitney test, p = 0.85).

### 3.3 Demethylation treatment restores SPARC mRNA levels in fibrotic UIP cell line

Since the epigenetic silencing occurred at promoter CpG island and was responsible for the downregulation of its expression in primary fibrotic and non-fibrotic cell lines, we explored and confirmed a different profile of *SPARC* methylation status in primary non-fibrotic (cell line 22) and one-fibrotic cell line (fibrotic UIP, FF24 cell line). QMSP analysis showed *SPARC* hypermethylation only in the fibrotic line.

To verify whether the repression of SPARC expression was correlated with CpG methylation at its promoter region, we examined the variation of SPARC mRNA level in non-fibrotic and fibrotic cell lines before and during treatment with 5-aza-dC at 24 h, 48 h and 72 h.

**FIGURE 4:**
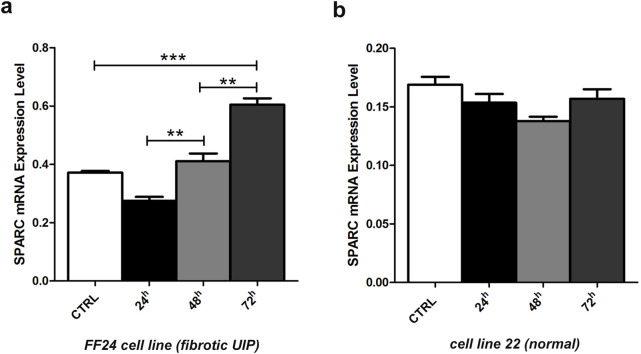
Changes in SPARC mRNA transcript levels in fibrotic and non-fibrotic cell lines. **(a)** Fibrotic FF24 cell line and **(b)** non-fibrotic cell line 22. Expression levels were evaluated by RT-qPCR before and after 5-aza-dC treatment at 24, 48, 72 h. Error bars indicate the standard deviation of three different experiments. **p < 0.01, ***p < 0.001, t-.test.

By RT-PCR analysis, a progressive rescue at SPARC transcript levels was observed after all tested time-points (24 h, 48 h, and 72 h), particularly evident after 72 h (t-test, p < 0.001) in fibrotic cells (Figure 4a), whereas SPARC expression did not reveal any significant variation in non-fibrotic cell (Figure 4b).

## 4 Discussion

The *SPARC* gene codifies a matricellular protein that is implicated in the regulation of tissue remodeling, cell proliferation and migration in lung cancer, chronic airway disease, and pulmonary fibrosis (Wong and Sukkar, 2017; Moretti et al., 2022). Its levels were found to decrease in normal adult tissues, while increasing in tissues with fast cell turnover, during embryonic development, and in response to damage, recognizing its crucial function in tissue repair and regeneration (Ghanemi et al., 2021). Among the several reported dysregulation mechanisms of SPARC expression, the aberrant methylation at the promoter region of *SPARC* gene has been widely reported in many solid tumors, such as endometrial, colon, pancreatic, ovarian cancer and lung cancer, and could lead to a worse clinical outcome in this group of patients (Fabrizio et al., 2020; Liu et al., 2018; Nagaraju and El-Rayes, 2013; Zhang et al., 2012). However, it remains un-investigated in lung fibrosis.

In our work, we sought to assess if aberrant methylation at the gene promoter region should be considered as one mechanism of SPARC dysregulation in lung fibrosis and if *SPARC* methylation correlates with IPF and/or NIPF.

As results, we observed significant and frequent hypermethylation at the *SPARC* promoter region in lung fibrotic tissues, when compared with non-fibrotic lung tissues. More specifically, *SPARC* aberrant methylation was detected more frequently in 45% (20/44, p-value <0.004) of IPF compared to NIPF 13% (3/23, p > 0.05). Considering that the overall *SPARC* methylation between two groups follows a different trend in terms of distribution and frequency, we confirmed that statistically significant methylation levels were evident in IPF, whereas this epigenetic event was not significantly reported in NIPF subgroup and in comparison, with NFLT tissues.

Concerning the discriminatory power of the *SPARC* methylation levels on FFPE tissues, noteworthy, an AUC of 0.68 with a threshold of 0.65 was obtained from the comparison between IPF and NFLT, confirming a sensitivity of 45% and a specificity of 100%. Nevertheless, it was not possible to retrieve SPARC protein expression from the public dataset and, at the same time, establish a possible correlation of SPARC expression with methylation levels in IPF and NIPF tissues of our cohort. However, this link was widely reported both at transcript and protein levels, both in cell lines and primary tumor tissues (Fabrizio et al., 2020; Liu et al., 2018; Li et al., 2022; Nagaraju and El-Rayes, 2013) and was also *in vitro* confirmed in our study, since SPARC expression was restored in the UIP lung cell line FF24 upon treatment with the demethylating agent 5-Aza-Cdr.

The intersection of epigenetic data available in the literature with those provided by our analysis in FFPE tissues and cell lines would allow us to suggest a new piece on the biological point of view of IPF disease. The first consideration is that a high SPARC expression profile is compatible with complex interactions between epithelial cells, endothelial cells, and stromal fibroblasts which are driven by epigenetic and signaling changes, exhibited distinct patterns during abnormal tissue remodeling, as well as in response to tissue injury and inflammation (Mei et al., 2021) generated by reactive oxygen species (ROS), (Veith et al., 2019). Secondly, IPF itself increases the risk of lung development and this link is also mediated by SPARC protein, by promoting microvascular remodelling and excessive deposition of ECM proteins, as well as by mediate paracrine epithelial-mesenchymal signaling (Wong and Sukkar, 2017; Ballester et al., 2019).

In this regard, among the multiple common genetic, molecular, and cellular processes that connect lung fibrosis with lung cancer, epigenetic variations have been hypothesized to predispose the patient to develop both IPF and lung cancer. CpG-island-specific DNA hypermethylation often occurs at the gene promoter region, which pushes the affected genes into an inactive state (Pfeifer, 2018). This epigenetic modification is believed to trigger the onset of pre-tumorous signals by increasing the risk of lung cancer patients’ progression (Stella et al., 2022). While *SPARC* promoter methylation is a frequent event in several cancers and may contribute to ECM dysregulation in both fibrotic and malignant settings, the extent to which this epigenetic mark confers increased cancer susceptibility in IPF remains unclear. This hypothesis could find confirmation in the multiple functions covered by *SPARC* gene as a tumor suppressor gene, since its silencing switch on a more malignant property in terms of cellular heterogeneity complexity (Said, 2016). Given the known epidemiologic link between IPF and lung cancer (Abu Qubo et al., 2022), and shared pathogenic features such as senescence, chronic injury, and aberrant epigenetic reprogramming (Zhan et al., 2025), it is plausible that *SPARC* silencing may participate in a common disease axis. However, these considerations remain speculative and warrant further investigation in longitudinal cohorts and functional cancer models (Geng et al., 2019).

To date, different questions and limitations of our study remain to be clarified. Firstly, the QMSP assay covers only 11 CpGs at the promoter region of the *SPARC* gene and does not allow us to measure the variation of methylation levels at single CpG in samples. As well-known, methylation does not affect all CpGs of specific promoter island, that were linked to different regulatory players of the epigenetic process (Li et al., 2014). These findings suggest that *SPARC* methylation alone may not provide sufficient diagnostic accuracy but could contribute meaningfully as part of a more comprehensive methylation panel. This approach aligns with the growing use of epigenetic panels that analyze multiple methylation sites in fibrotic lung disease, enhancing both sensitivity and specificity. There is a clear need to explore integrative models to improve clinical applicability and diagnostic accuracy. Future studies employing high-resolution approaches, as well as pyrosequencing or targeted bisulfite sequencing, are warranted to accurately map methylation at individual CpG sites within the *SPARC* promoter, overcoming the limitations of QMSP (Consortium, 2016), and to elucidate their regulatory role in transcriptional silencing mechanisms associated with IPF.

Anyhow, we demonstrated that variations in methylation level at promoter region detected by our assay are functional. As shown, with the use of 5-Aza-CdR, the expression of SPARC in pulmonary fibrosis cells was modulated. While our cell line-based analyses provide preliminary functional evidence for methylation-driven silencing of *SPARC*, the need to incorporate a comprehensive panel of primary fibroblasts derived from both IPF patients and healthy donors will be essential to assess interindividual variability and confirm reproducibility of these findings. Secondly, the scoring of SPARC protein expression by immunohistochemical (IHC) analyses was not established.

Data about a possible link between *SPARC* methylation and protein levels are available for tumors, but they should not be always homogeneous, since protein levels in cell should be also linked to post-transcriptional controls (Chen et al., 2014; Liu et al., 2018). While we demonstrate SPARC mRNA reactivation following 5-Aza-CdR treatment in fibrotic cell lines, which supports epigenetic silencing via promoter methylation, additional studies are needed to include IHC assays on FFPE lung tissues in order to validate whether *SPARC* methylation correlates with protein repression.

Moreover, given the established influence of age, smoking, environmental exposures, and other clinical variables on DNA methylation, future studies should incorporate multivariate regression models to adjust these covariates, thereby enabling more precise screening and identification of disease-specific epigenetic signatures, including the assessment of *SPARC* methylation as an independent biomarker for IPF.

Finally, a correlation analysis between *SPARC* methylation levels, protein expression, clinical-pathological features and disease outcomes in IPF patients is warranted in larger and independent datasets.

## 5 Conclusion

Given the growing body of evidence linking SPARC to respiratory disorders, it is also critical to investigate potential treatment targets for this molecule (Conforti et al., 2020). At this time, SPARC cannot be pharmacologically inhibited. In any case, methods that take advantage of *SPARC*’s epigenetic regulation may also be able to therapeutically modify its expression in particular illness situations. DNA methylation changes have been shown to drive tumor formation and malignant progression and as such have established basic mechanisms for disease pathogenesis, as well as targets for intervention in cancer (Sgalla et al., 2018).

Despite the many existing challenges, epigenomic profiling to understand the dynamic biology of IPF aims to discover new biomarkers that find application in diagnosis and prediction of treatment response for this group of patients (Bartold et al., 2024). Actually, the link between this epigenetic event and specific IPF histological patterns remains to be fully established and requires further investigation (Wells et al., 2018).

The latest, intriguing findings reported here on *SPARC* methylation may suggest its potential as a valuable clinical application.

Future research is essential to deepen the molecular pathogenesis of IPF and investigate potential therapeutic strategies to meet the unmet needs of patients living with a diagnosis of IPF.

## Data Availability

The original contributions presented in the study are included in the article/Supplementary Material, further inquiries can be directed to the corresponding authors.

## In memoriam

This manuscript is dedicated to Dr. Giulio Rossi, who sadly passed before the manuscript publication.
