# Supplementary material for: SPARC aberrant methylation in idiopathic pulmonary fibrosis: an explorative study
Source: Front Cell Dev Biol. 2025 Sep 3;13:1639844. doi: 10.3389/fcell.2025.1639844 (PMC12440879; doi:10.3389/fcell.2025.1639844)
Supplement: Supplementary file 1 [file DataSheet1.docx]

Supplementary Material

# Supplementary Figure

**
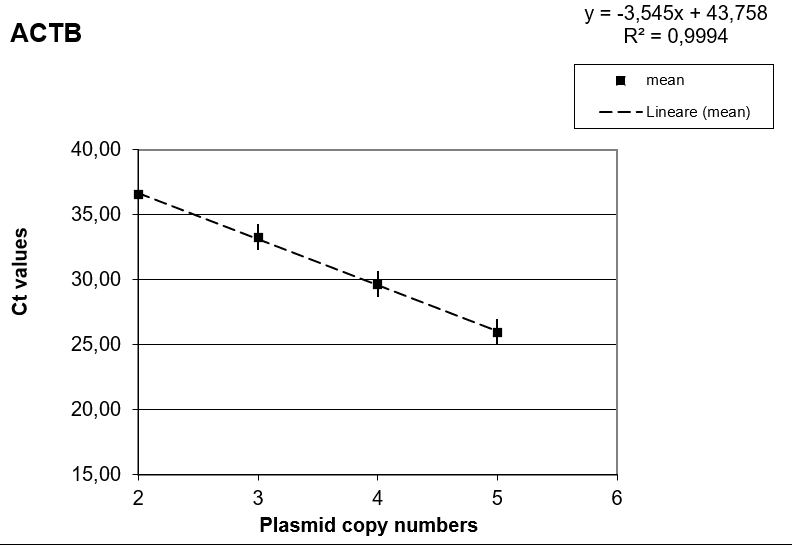
**

**
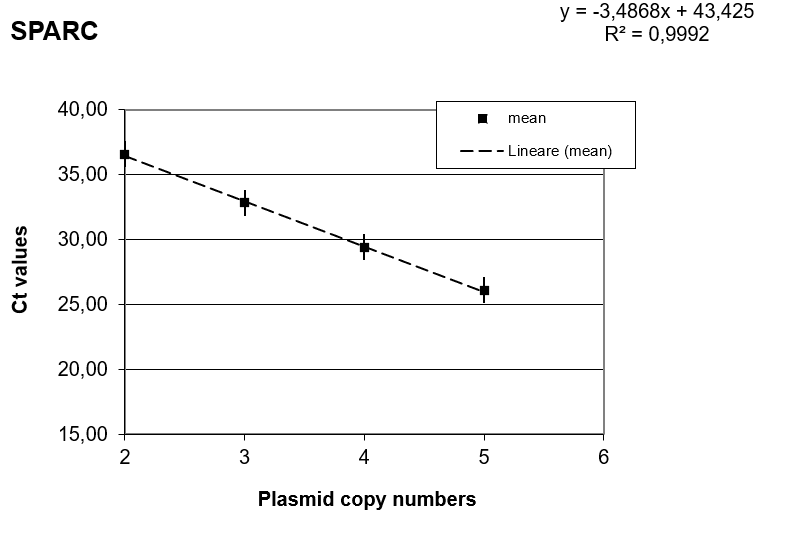
**

**Supplementary Figure 1.** **Standard curves for *ACTB* (A) and *SPARC* (B).** The panels show the mean of Ct values versus log of plasmid copy numbers as triplicate. Slope and intercept are represented in the equations of the regression lines, together with regression coefficient.

# Supplementary Tables

**Supplementary Table 1.** AUC values and cut-off values in IPF, NIPF and NFLT tissues.

| Histology | AUC | Cut-off | Sens | Spec | p-value MW |
| --- | --- | --- | --- | --- | --- |
| NIPF vs NFLT | 0,49 | 1,30 | 0,13 | 1,00 | 0,85780 |
| IPF vs NFLT | 0,68 | 0,65 | 0,45 | 1,00 | 0,04219 |
| NIPF+IPF vs NFLT | 0,61 | 0,65 | 0,34 | 1,00 | 0,1553 |
| NIPF vs IPF | 0,68 | 0,35 | 0,45 | 0,87 | 0,004643 |

AUC, Area under the ROC curve; sens, sensitivity; spec, specificity; NIPF, non-idiopathic pulmonary fibrosis; NFLT, non-fibrotic lung tissues; IPF, Idiopathic pulmonary fibrosis. p-value MW, Mann Whitney test.

**Supplementary Table 2**. Summarized boxplots values for DNA methylation levels.

| Histology | mean | SD | median | min | q1 | q3 | max |
| --- | --- | --- | --- | --- | --- | --- | --- |
| NIPF | 0,3900 | 1,0712 | 0,0000 | 0,0000 | 0,0000 | 0,0000 | 3,9500 |
| IPF | 5,5473 | 18,0591 | 0,0000 | 0,0000 | 0,0000 | 5,8250 | 118,99 |
| NFLT | 0,0764 | 0,1880 | 0,0000 | 0,0000 | 0,0000 | 0,0000 | 0,6000 |

SD, standard deviation; min, minimum; q1-q3, quartile 1-3; max, maximum; NIPF, non-idiopathic pulmonary fibrosis; IPF, Idiopathic pulmonary fibrosis; NFLT, non-fibrotic lung tissues
